# Research progress of T cell autophagy in autoimmune diseases

**DOI:** 10.3389/fimmu.2024.1425443

**Published:** 2024-07-22

**Authors:** Xingxing Zhao, Dan Ma, Baoqi Yang, Yajing Wang, Liyun Zhang

**Affiliations:** Third Hospital of Shanxi Medical University, Shanxi Bethune Hospital, Shanxi Academy of Medical Sciences, Tongji Shanxi Hospital, Taiyuan, China

**Keywords:** T cells, autophagy, pathogenesis, autoimmune disease, systemic lupus erythematosus, rheumatoid arthritis

## Abstract

T cells, as a major lymphocyte population involved in the adaptive immune response, play an important immunomodulatory role in the early stages of autoimmune diseases. Autophagy is a cellular catabolism mediated by lysosomes. Autophagy maintains cell homeostasis by recycling degraded cytoplasmic components and damaged organelles. Autophagy has a protective effect on cells and plays an important role in regulating T cell development, activation, proliferation and differentiation. Autophagy mediates the participation of T cells in the acquired immune response and plays a key role in antigen processing as well as in the maintenance of T cell homeostasis. In autoimmune diseases, dysregulated autophagy of T cells largely influences the pathological changes. Therefore, it is of great significance to study how T cells play a role in the immune mechanism of autoimmune diseases through autophagy pathway to guide the clinical treatment of diseases.

## 1 Introduction

Adaptive immune responses mediated by T cells and B cells are essential for protective immunity against pathogens, and T cells not only activate and proliferate B cells through surface co-stimulatory molecules and secreted cytokines, but also assist in the formation of germinal centers (GCs), the differentiation of B cells into plasma cells and memory B cells, and the production of a large number of autoantibodies (1). Moreover, T cells play a role in the early stage of the immune response through a variety of mechanisms such as antigen recognition, activation, differentiation, secretion of cytokines and regulation of the immune response. Therefore, T cells play a more important role in the early stage of autoimmune diseases, and an in-depth understanding of the mechanism of T cells in autoimmune diseases can help provide ideas for the treatment of the disease.

Autophagy was first reported to occur under conditions of nutrient stress, resulting in the generation of small molecules and energy to promote cell survival. Autophagy is a lysosomal-mediated catabolic process that maintains cell homeostasis by degrading damaged cytoplasmic components and organelles (2). In general, autophagy has a protective role, enabling cells to adapt to internal and external stresses such as nutrient deprivation, oxidative stress, hypoxia, DNA damage, and the accumulation of damaged proteins and organelles in cells (3). Autophagy can remove intracellular pathogens, damaged organelles, and misfolded proteins, participate in host protection, and play a key role in the development and function of the immune system and immune cells. Autophagosomes have been found in both human and mouse T cells (4, 5). And autophagy has been observed to play a critical role in the development, survival, proliferation, and differentiation of T cells (6).

It has been demonstrated that dysregulation of autophagy is associated with many diseases, including neurodegenerative diseases, infections, inflammation, metabolic dysfunction, cancer and aging (7). An increasing number of studies have indicated that immune disorders in autoimmune diseases are associated with abnormal regulation of autophagy, including host defense against various pathogens, unconventional secretion of cytokines, and antigen presentation. Therefore, it is necessary to further explore the effects of autophagy on autoimmune diseases through the regulation of T cells in this paper and to discover effective strategies and therapeutic targets for suppressing autoimmune responses through the autophagy pathway.

## 2 Autophagy

### 2.1 Classification of autophagy

Autophagy is traditionally regarded as a bulk, non-selective process that indiscriminately sequesters and degrades cytoplasmic components, including organelles and macromolecular complexes. This occurs in response to nutrient starvation, commonly referred to as bulk autophagy (8). However, it is gradually recognized that autophagy operates in a highly selective manner, in which it degrades specific targets, usually potentially harmful cargo. Selective autophagy encompasses a range of processes that target specific cargo, including damaged organelles (mitophagy, lysophagy, ER-phagy, ribophagy, pexophagy), aggregated proteins (aggrephagy) and invading bacteria (xenophagy). These autophagy play an essential role in maintaining cellular quality control (8).

Autophagy is divided into three main categories: macroautophagy, microautophagy, and chaperone-mediated autophagy (CMA). These pathways differ in the manner by which cellular material is transported to lysosomes. Macroautophagy (also known as autophagy) involves the engulfment of a large number of cytoplasmic substances, organelles, protein aggregates, and microorganisms through the formation of autophagosomes, which are then degraded after fusion with lysosomes (9). This is the most common form of autophagy. Microautophagy refers to the direct encapsulation of proteins and small organelles into lysosomes for degradation. CMA necessitates the recognition of substrate molecules by a constitutively expressed cytoplasmic heat shock protein, HSPA8 (also known as HSC70), and the resulting complex is transferred to lysosomes for degradation via lysosomes-associated membrane protein 2A (LAMP2A) (10, 11). Among these, macroautophagy and microautophagy can engulf structures with large molecular weight through selective and non-selective mechanisms, while CMA can only degrade part of soluble proteins (12). Although the three methods are different, they are all designed to enable cells to adapt to environmental pressure and maintain the stability of the intracellular environment.

### 2.2 Process of autophagy

**Figure 1:**
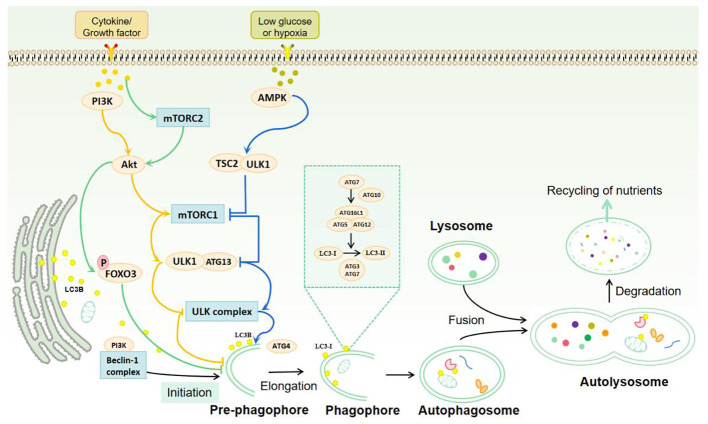
Autophagy process and signal regulation. Autophagy is mainly induced by mTOR complex, ULK1 complex, Beclin-1 complex and protein kinase (AMPK). Under the stimulation of cytokines or growth factors, mTORC mechanical targets are activated to reverse the inhibition of ULK1 complex and the initiation of autophagy. AMPK is activated under hypoglycemic or hypoxic conditions to further stimulate the ULK1 complex and Beclin-1 complex, thereby inducing autophagy. The process of autophagy includes induction, elongation, maturation, fusion and degradation. mTOR and AMPK transmit stress signals through the ULK1 complex to promote autophagy initiation. Beclin-1 interacts with autophagy proteins and mediates the formation of the double-layer membrane structure of the autophagosome. Atg7 and Atg10 induce the binding of the Atg5-Atg12 complex to Atg16L1 and promote the binding of LC3-II to the endosomal and outer membranes of the phagosome. LC3B forms cytoplasmic LC3-I in the presence of Atg4. With the help of Atg5-Atg12/Atg16L1 complex, LC3-I is lipified by Atg3 and Atg7 to membrane-bound LC3-II, which mediates autophagosome elongation and autophagosome formation. Mature autophagosomes fuse with lysosomes to form autolysosomes. The acidic environment of lysosomes and hydrolases degrade the inner membrane and contents of autophagosomes, and the resulting substances are released back to the cytoplasm to participate in cell life activities and self-renewal of cells.

The process of autophagy is comprised of five distinct stages: induction, elongation, maturation, fusion, and degradation. These stages are regulated by the autophagy-related gene (Atg) family. Currently, 31 yeast Atg genes and more than 18 mammalian Atg gene homologs have been confirmed to be related to autophagy. These include Atg1/ULK1, Atg6/Beclin1, Atg8/LC3, and others (13). In the induction stage of autophagy, the mammalian target of rapamycin (mTOR) and adenosine 5’-monophosphate-activated protein kinase (AMPK) transmit stress signals through the downstream effect of the unc-51-like kinase 1 (ULK1) complex. In response to certain stress, the ULK1 complex initiates autophagy and induces the formation of autophagy precursors (14). In the initiation stage of autophagy, the Beclin1/hVps34 complex interacts with autophagy proteins to mediate the formation of the autophagy precursor bilayer structure. Its activity is regulated by type III phosphatidylinositol 3-kinase (PI3K) (15). In the elongation stage of autophagy, the Atg5-Atg12 complex is formed by the action of Atg7 and Atg10, which then dimerises with autophagy-associated protein 16-1 (Atg16L1). This complex subsequently transfers to the initiation site of autophagy, where it promotes the binding of LC3-II to the inner and outer membranes of phagosomes, thus facilitating membrane recombination. In the presence of the cysteine protease Atg4, microtubule-associated protein light chain 3 (LC3B) forms cytoplasmic type LC3-I. With the help of Atg5-Atg12/Atg16L1 complex, LC3-I is liposomalised by Atg3 and Atg7 to membrane-bound LC3-II (16). At the stage of autophagy maturation, fusion and degradation, the majority of autophagic proteins are shed from their outer membrane following autophagosome closure. The mature autophagosome then fuses with lysosomes to form the autophagolysosome. Subsequently, the acidic environment of lysosomes and hydrolases degrade the inner membranes and contents of autophagosomes, releasing the produced substances back to the cytoplasm to re-participate in cellular life activities and achieve self-renewal of the cells (17) (**Figure 1**).

The enriched PI3P structure in the endoplasmic reticulum (ER) is considered to be the site of autophagogenesis and the main source of autophagosomes, releasing LC3, which is required for autophagosome formation (18). LC3-II is considered to be an important marker of autophagy, and the conversion of LC3-I to LC3-II is an essential process during autophagosome maturation. Another important marker of the autophagic process is nucleus-derived p62/SQSTM1. p62 is a selective autophagy receptor, and one of its key roles is to bind to LC3-II and polyubiquitinated proteins, delivering the bound ubiquitinated proteins to autophagosomes, ultimately leading to their degradation by lysosomes (19). The transcription of p62 is modulated by the conserved autophagy and lysosomal regulatory transcription factor EB (TFEB) (20). The term autophagic flux is used to describe the complete catabolic process that ensures the decomposition of materials to be degraded and the release of macromolecules in the cytoplasm (21). This process is an indicator of autophagic activity.

### 2.3 Regulation of autophagy

The initiation and maturation of autophagosomes is regulated by multiple factors in a bidirectional manner. These include the serine/threonine protein kinase ULK1 complex, the Beclin1 complex, and AMPK, which act to stimulate autophagy. Conversely, mTOR and its associated complexes, the mechanistic target of rapamycin complex 1 (mTORC1) and mTORC2, exert a negative regulatory effect on autophagy (22). Furthermore, insulin and other growth factor signals activate type I PI3K-Akt, which inhibits autophagy by activating mTORC1 and inhibiting Beclin1, type III PI3K complex, and Atg14L (23).

#### 2.3.1 mTOR signaling pathway

The highly conserved serine/threonine kinase mTOR belongs to the phosphatidylinositol 3-kinase-related kinase (PIKK) family, which regulates signaling pathways related to cell growth through phosphorylation (24). mTOR signaling pathway is one of the main mechanisms of autophagy regulation and also the main inhibitory pathway of autophagy (24). Under environmental signals such as starvation, autophagy can be inhibited by inhibiting mTOR (2). The growth factor/PI3K/Akt signaling pathway is involved in the upstream regulation of mTOR. Protein kinase B (Akt) is phosphorylated by PI3K, and PI3K/Akt further phosphorylates mTOR (25, 26). Activated mTOR can not only accelerate the cell cycle but also inhibit the occurrence of autophagy (27). Therefore, the activation of the PI3K-Akt-mTOR signaling pathway negatively regulates autophagy, which is essential in preventing autophagy induction (**Figure 1**).

mTORCl is primarily involved in the regulation of cell development, apoptosis, energy metabolism, and autophagy (28). AMPK and mTORC1 are sensors of cellular energy production and energy metabolism *in vivo* and are the two major regulatory units that control autophagy (29). With sufficient nutrient availability, mTORC1 kinase integrates signals from nutrients and growth factors and acts as a junction for cellular signals to control growth and protein synthesis (30). In the absence of physiological stressors, mTORC1 activation inhibits autophagy by phosphorylating ULK1 and Atg13 (31). Conversely, in the context of nutrient deficiency, mTORC1 activity is inhibited, resulting in ULK1 and Atg13 dephosphorylation. This dephosphorylation enables the ULK kinase complex to become activated, thereby inducing autophagy (32). Energy deficiency can also activate AMPK, which is involved in ULK1 activation and phosphorylation of tuberous sclerosis protein 2 (TSC2). Both of these processes inhibit mTORC1 activity and promote autophagy activation (33) (**Figure 1**).

Although mTORC2 is primarily associated with the formation of cytoskeletal proteins and the regulation of cell survival, some studies have identified its involvement in the process of autophagy (34). mTORC2 is a bidirectional regulator of autophagy. On the one hand, mTORC2 inhibits autophagy indirectly through the Akt/Forkhead box protein O3 (FOXO3) axis. FOXO3 is a transcription factor that is both necessary and sufficient for the induction of autophagy. In the context of nutrient-rich conditions, mTORC2 activates Akt by phosphorylating Akt, leading to phosphorylation and inactivation of FOXO3. This results in the retention of FOXO3 in the cytoplasm and the loss of its transcriptional activity, which in turn reduces the transcription of autophagy-related genes LC3 and Bnip3. Ultimately, this inhibits the induction of autophagy (35). During periods of nutrient starvation, FOXO3 undergoes dephosphorylation and translocalization to the nucleus, where it activates autophagy-related genes to induce autophagy (36). This effect is not prevented by rapamycin. The mTORC2/Akt/FOXO3 signaling pathway has a crucial role in the suppression of autophagy. Conversely, it has been proposed that mTORC2 is implicated in the regulation of microfilament cytoskeleton and endocytotic pathways, and that mTORC2 plays a pivotal role in the maturation and transportation of autophagy vesicles (37).

#### 2.3.2 Beclin1 signaling pathway

Beclinl is composed of a Bcl-2 binding site (BH3), an evolutionarily conserved domain (ECD), and a helic-helical domain (CCD) (38). Studies have confirmed that autophagy regulatory proteins can directly or indirectly bind to different domains of Beclin1 to form new protein complexes, paving the way for the regulation of autophagy (39). In addition. The BH3 receptor of Bcl-2/Bcl-XL can also inhibit autophagy by competitively binding with Beclin1 protein and blocking the formation of the complex. In contrast, phosphorylation of BH3 by the death-related protein kinase gene (DAPK) can reduce the binding force of Beclin1 and Bcl-XL, thus enhancing autophagy (40). The Beclin1 gene can combine with type III P13K through its CCD and ECD domains, thereby promoting the formation and maturation of the autophagosome membrane and participating in the initiation of autophagy (41).

## 3 The regulation of autophagy on T cells

Autophagy represents an essential catabolic process in T cell biology, regulating T cell development, activation, proliferation, and differentiation by regulating cell metabolism (6).

### 3.1 Effects of autophagy on T cell development

During central tolerance, thymic epithelial cells (TECs) present self-antigen-derived epitopes on MHC class II molecules, thereby ensuring the generation of self-tolerant CD4^+^ T cells. Autophagy is able to process certain self-ligands presented by MHC-class II and participate in positive and negative T cell selection in the thymus, thereby promoting the generation of a self-tolerant T cell repertoire (42).

Bone marrow hematopoietic stem cells (HSC) are highly capable of self-renewal and differentiation. Autophagy plays a pivotal role in the development and differentiation of mature T lymphocytes in HSCs. HSCs with autophagy gene deletion exhibit a decreased self-renewal ability and a greater tendency to differentiate into myeloid stem cells, resulting in abnormal proliferation of myeloid cells in the bone marrow, a decreased proportion of lymphoid cells, a large number of mitochondria in cells, and a significant increase in reactive oxygen species (ROS) levels. Autophagy can remove abnormally accumulated mitochondria in a timely manner to maintain the normal function of HSCs. Consequently, the normal functioning of autophagy is a prerequisite for the generation of lymphoid progenitor cells (43).

### 3.2 Effect of autophagy on T cell activation

In TECs, autophagy plays an important role in the activation of T cells. The autophagy activity of T cells remains at a low level in the resting state but is significantly upregulated after activation. The activation of T cells is regulated by antigen presentation by antigen-presenting cells (APC) expressing MHC-II class and significantly affects peripheral T cell proliferation (44–46). T cell activation is dependent on the stable calcium flow within the ER. Autophagy plays a role in T cell activation by maintaining the homeostasis of calcium flow within the ER (47, 48). At the same time, autophagy degrades proteins, lipids, and glycogen, providing energy substrates for T cell activation. Inhibition of this catabolism prevents effective T cell activation.

### 3.3 Effect of autophagy on T cell proliferation

In activated T cells, numerous autophagy genes (including Atg5, Atg7, Atg8, and Beclin1) were found to be upregulated, promoting autophagosome formation (49). However, in a conditional knockout (CKO) mouse model, effector T cells specifically knocked out of autophagy genes (e.g., Atg3, Atg5, Atg7, Atg16L1, Beclin1) showed reduced cytokine secretion and cell proliferation upon activation (49). For example, decreased thymocytes and peripheral lymphocytes and increased CD8^+^ T cell death are observed in Atg5^-/-^ bone marrow chimeric mice. These Atg5^-/-^ T cells demonstrated a lack of proliferation in response to T cell receptor (TCR) stimulation (50). Atg7^-/-^ T cells from CKO mice show increased cell death, increased content of the endoplasmic reticulum, and increased number of mitochondria (48). Similar results have been observed in Atg3^-/-^ CKO mice, which exhibited impaired autophagy (51). Furthermore, Foxp3^+^ T cell-specific knockout of Atg5 or Atg7 decreases the stability and survival of regulatory T cells (Treg cells) (52). Consequently, autophagy is essential for the survival and development of T cells in an activated environment and for their functional integrity.

### 3.4 Effect of autophagy on T cell differentiation

Cytokines and their receptors play an important role in regulating T cell differentiation and function. In fact, the differentiation of each CD4^+^ T subset is driven by a specific cytokine. For instance, IL-2, a common γ-chain cytokine, is secreted early after TCR action on CD4^+^T cells, induces autophagy in CD4^+^T cells, and plays a role in the ongoing proliferation and effector differentiation of the cells as well as in the formation of memory cells (53). As CD4^+^ T cell subsets utilize different cytokine signaling pathways and their respective metabolic pathways, autophagy also differentiates and functions to different degrees (54, 55). For example, autophagy inhibits the expansion and differentiation of Th2, Th9, and Th17 to attenuate inflammatory responses, whereas Th1 and Treg are dependent on autophagy for differentiation and function (56, 57). IL-21 produced by Tfh cells induces mTOR activation, promotes differentiation of naïve T cells into helper T (Th cells) cells (Th1, Th2, Th9, and Th17), and inhibits autophagy and differentiation of Treg cells (58–64).Conversely, when the level of mTOR activation is low and the level of AMPK is high, naïve T cells exhibit a preference for differentiation into Treg cells. Although all Th cells rely on mTOR activity, Th1 and Th17 cells require Rheb-dependent mTORC1 activation, whereas Th2 differentiation is activated by mTORC2 (59). It has been demonstrated that the induction of autophagy in bone marrow-derived dendritic cells (DCs) results in a significant proliferation of T follicular helper (Tfh) cells and the induction of an inflammatory response (65). However, the precise mechanisms by which autophagy regulates the proliferation, differentiation, and function of Tfh cells under different physiological and environmental conditions remain to be elucidated. Autophagy has also been shown to promote the proliferation of CD8^+^ T cells and the generation of CD8^+^ memory T cells (66). However, there are fewer studies related to autophagy on CD8^+^ T cells, and the role of autophagy in memory T cells and cytotoxic T cells is not yet fully determined.

### 3.5 Regulation of autophagy in the metabolism of T cells

In the context of nutrient limitation or other forms of stress, autophagy plays a crucial role in enabling T cells to adapt to environmental challenges and maintain their functionality. This process involves the provision of substrates essential for the synthesis of new cellular components and the acquisition of energy, as well as the regulation of metabolic pathways within T cells. In activated T cells, autophagy has been demonstrated to regulate metabolic pathways to modulate cellular function and differentiation (67). Atg7-deficient CD4^+^ T cells exhibit reduced ATP production in response to TCR engagement, and both anaerobic glycolysis and mitochondrial respiration are reduced when lysosomal activity is blocked, suggesting that inhibition of autophagy’s catabolic processes may prevent effective T cell activation (68). A novel mechanism has recently been proposed to support the ability of autophagy to regulate T cell metabolism through activation of mTORC1. In activated T cells, the bridging protein TAX1BP1 promotes autophagy-mediated degradation of cytoplasmic proteins to generate L-cysteine, which activates mTORC1 to sustain the activation-induced response and drive the metabolic changes necessary for cell proliferation (69). Consequently, CD4^+^ T cells lacking TAX1BP1 are unable to initiate glycolytic anabolic changes to meet the energy demands of activation and exhibit reduced expression of hexokinase 2 and Glut1. Interestingly, starvation-induced autophagy did not appear to be affected, which seems to support the characterization of increased autophagic activity upon T cell activation (69).

### 3.6 Autophagy and T cell homeostasis

The absence of autophagy-related proteins impairs the degradation of damaged organelles in T cells, resulting in the accumulation of damaged mitochondria, an increase in reactive oxygen species, and ultimately disrupting T-cell homeostasis (70). FOXP3 is an important transcription factor for the control of autoimmune responses in Treg cells. Studies have demonstrated that the autophagy-associated protein AMBRA1 enhances the stability of the transcriptional activator FOXO3 by interacting with the phosphatase PP2A, which in turn triggers FOXP3 transcription, resulting in an increase in the number of Treg cells. This evidence suggests that autophagy plays an essential role in regulating the dynamic homeostasis of Treg cells (71). Furthermore, it was demonstrated that mice with a deletion of the T cell-specific activated protein C-kinase receptor 1 (RACK1) exhibited a notable reduction in the number of peripheral CD8+ T cells or CD4+ T cells, accompanied by a blockage in mitochondrial degradation. This suggests that the deletion of RACK1 resulted in autophagy inhibition, which ultimately led to mitochondrial accumulation and the disruption of CD4+ T cell and CD8+ T cell homeostasis (72). Consequently, RACK1 may be a pivotal regulator in the control of T cell homeostasis.

### 3.7 Epigenetics of T-cell autophagy

Although the mechanisms of autophagy in response to starvation, hypoxia, or receptor stimulation have been extensively studied, the critical epigenetics that initiate and maintain the autophagic process remain unknown. Epigenetics refers to the study of reversible and hereditable phenotype changes in gene expression without altering genetic or DNA sequence, such as chromatin remodeling and protein modification (73). Histone methyltransferase G9a is responsible for coordinating the transcription and activation of key regulators of autophagosome formation by remodeling the chromatin. In normal conditions, G9a binds to LC3B, WIPI1, and DOR gene promoters and inhibits gene expression in a methyltransferase-dependent manner (74). However, during the activation of starved or receptor-stimulated naïve T cells, G9a and its repressive histone mark are removed from these promoter loci, allowing the expression of these genes to maintain autophagy (74). Pharmacological inhibition or RNA interference (RNAi)-mediated suppression of G9a resulted in increased LC3B lipidation and expression, increased p62 aggregation, and the formation of autophagosomes (75). These findings collectively demonstrate that G9a exerts a direct inhibitory effect on autophagy gene expression. Moreover, the inhibition of G9a-mediated epigenetic repression emerges as a pivotal regulatory mechanism during autophagy.

Protein modification refers to the chemical modification of proteins after their biosynthesis, which is also called post-translational modification (PTM). PTM includes phosphorylation, acetylation, citrullination, and methylation, and so forth (35). PTMs are widely involved in the regulation of autophagy. For example, phosphorylation in autophagy regulates the activity of autophagy-associated proteins and the initiation and progression of autophagy by regulating signaling pathways. Acetylation and deacetylation are both involved in the regulation of autophagy initiation and selective autophagy by controlling the acetylation level of important proteins in the autophagy process.

Peptidylarginine deiminase type 2 (PADI2) catalyzes the conversion of arginine residues on proteins to citrulline residues, which results in protein unfolding and loss of function. In activated Jurkat T cells, PADI2 regulates T cell activation and Th17 cytokine production. Overexpression of PADI2 significantly increases the level of citrullinated proteins and induces unfolded protein response (UPR) signaling and ROS production, which ultimately promotes autophagy and leads to the early degradation of p62 and accumulation of LC3B-II (76). Autophagy, the designated mechanism for unfolded protein degradation, is involved in the generation of citrullinated peptides by APCs (77). Consequently, citrullination represents the “tag” of protein end-of-life and degradation, which is related to cell survival mechanisms and autophagy.

Future research will aim to identify the epigenetic changes that drive gene expression, further characterize transcription factors and other epigenetic modifiers, provide insight into how these genes are specifically regulated during autophagy induction, and reveal potential therapeutic targets to enhance or inhibit autophagy induction.

## 4 The pathogenesis of T-cell autophagy in autoimmune diseases

Autophagy is involved in both innate and acquired immune responses and plays a key role in anti-microbial interactions, major histocompatibility complex (MHC)-presented antigen processing, and lymphocyte development, survival, and proliferation (78). Under normal circumstances, autophagy in cells is tightly controlled. However, in autoimmune and degenerative joint diseases, autophagy dysfunction is largely responsible for pathological changes.

### 4.1 Systemic lupus erythematosus

SLE is an autoimmune disease that is characterized by the excessive production of autoantibodies, the deposition of immune complexes, the persistent production of type I interferons (IFN), the dysregulation of T cell responses, and the persistent inflammation (79). Autophagy is considered to be the core pathogenic factor of SLE abnormal immunity, affecting innate and acquired immunity. The factors contributing to the increased risk of SLE are not limited to single nucleotide polymorphisms (SNPs) but include several autophagy-related genes such as Atg5 (80), Atg7 (80), LRRK2 (81), IRGM (80), DRAM1 (82), CDKN1B (82), MTMR3 (83), and APOL1 (84). Several SNPS in the Atg5 gene have been found to confer genetic susceptibility to SLE from genome-wide association studies (GWAS) (80). And one SNP, rs573775, was associated with IL-10 production and a higher risk of SLE (85).

The alteration of T lymphocyte homeostasis is considered to play a key role in the pathogenesis of SLE (86). In SLE, hyperactivation of T cells due to enhanced cytokine-induced and autoantigen-induced signaling manifests as increased phagocytic vacuoles (87, 88), which may be associated with dysfunction of mitophagy. The degradation disorder of hypoxia-inducible factor (HIF)2α in SLE prevents the activation of the ubiquitin-proteasome system (UPS) upstream of mitophagy, resulting in the reduction of mitophagy and excessive accumulation (89). However, the specific mechanisms by which abnormal mitochondrial autophagy affects T-cell immunity need to be further investigated.

Studies showed that autophagy-related genes, including mTOR, Beclin1, LC3, and p62, were differentially expressed in the peripheral blood mononuclear cells (PBMC) of SLE patients (90). It was further observed that the LC3-II levels of CD4 and CD8 memory T cells isolated from peripheral blood of healthy individuals were significantly higher than those of naïve T cells. In SLE patients, this difference was observed in the CD8 subset but not in the CD4 subset. This suggests that autophagy may play an active cytoprotective and homeostatic role in rapidly proliferating activated T cells (49). Notably, naïve CD4^+^ T cells in the peripheral blood of SLE patients exhibited elevated levels of LC3-II and exhibited significantly higher levels of autophagy than those of healthy individuals. In contrast, there was no significant difference in autophagy levels of naïve and memory T cells in the CD4 memory T cell and CD8 subpopulations (91) (**Table 1**). In a separate analysis of T lymphocyte gene expression, it was observed that T lymphocytes cultured in the presence of serum from SLE patients exhibited negative regulatory proteins for autophagy. The expression of α-synuclein (SNCA), v-akt murine thymoma viral oncogene homolog 1 (Akt1), nuclear factor of κ light polypeptide gene enhancer in B-cells 1 (NFκB1) and B-cell lymphoma 2 (Bcl2) is specifically elevated in the presence of serum from SLE patients (105–108). Among these genes, SNCA appears to be the most prominent, as overexpression of this gene has been shown to inhibit autophagy by reducing autophagosome formation at an early stage. Akt1 and NFκB1 inhibit autophagy by activating the upstream inhibitor of autophagy, mTOR, whereas Bcl-2 inhibits autophagy through the inhibition of Beclin1. It is hypothesized that autophagy may be important in the early stages of T cell activation when extracellular nutrients are limited but metabolic reprogramming for proliferation has already begun. It is postulated that activated autophagy can meet the energy and material requirements for activation. It is postulated that CD4^+^ T cells may be activated at an earlier stage than autophagy in CD8^+^ T cells (109, 110). Additionally, elevated levels of autophagy in T cells were observed in the peripheral blood of lupus mice, with a positive correlation between disease severity and autophagy levels (92). Injection of shAtg5 lentivirus into lupus-susceptible mice resulted in decreased autophagy levels in peripheral blood lymphocytes, decreased numbers of lymphadenopathy, plasma cells, CD4^-^CD8^-^ and CD4^+^ T-cells, proteinuria, serum anti-dsDNA antibodies, B-cell activating factor (BAFF) and glomerular immune complex deposition levels were reduced, and lupus symptoms improved (92). Other similar studies have demonstrated that CD4^+^ and CD8^+^ T cells from Atg5^-/-^/Atg7^-/-^/Atg3^-/-^ CKO mice stimulated with TCR exhibited deficient proliferation, increased cell death, increased mitochondrial and endoplasmic reticulum content, and altered calcium flux after TCR stimulation (48). Although the precise mechanism underlying the enhanced autophagic activity observed in SLE is yet to be elucidated, evidence suggests that dysregulation of this pathway may contribute to the survival and proliferation of autoreactive T cells and the production of autoantibodies, which in turn exacerbate lupus. Furthermore, inhibition of autophagy has been demonstrated to be an effective approach in ameliorating lupus symptoms. Further understanding the specific mechanisms of autophagy dysregulation in T cell regulation in SLE will help us to develop effective targets and therapeutic approaches for inhibiting T cell autophagy.

**Table 1:** Regulation of autophagy on T cells and autoimmune diseases.

Diseases	Methods	Cell types	Autophagylevel	Effects
SLE [91]	\	CD4^+^ T cells (human PB)	Increased	LC3-II increased; There were no autophagic differences in CD8 subsets.
SLE [48, 92]	shAtg5-lentivirus mice Atg7^f/f^ Lck-Cre mice	lymphocytes (PB)	Decreased	The number of plasma cells, CD4^−^CD8^−^, CD4^+^ and CD8^+^ T cells were reduced. Albuminuria, serum anti-dsDNA antibodies, BAFF, and glomerular immune complex deposits were reduced, and lupus symptoms were improved.CD4^+^ and CD8^+^ T cells increased cell death, mitochondria and endoplasmic reticulum content, calcium flux change.
SLE [93, 94]	Treatment with CQ/HCQ	Th17 and Treg cells (mice PB)	Decreased	The levels of Th17 cells, IL-17, serum INF-γ, anti-dsDNA IgG, and anti-nuclear IgG were decreased; Treg cells and TGF-β were increased.
RA [95]	\	Naïve CD4^+^ T cells (human PB)	Decreased	The production of PFKFB3, ATP and ROS decreased; T cells sensitive to apoptosis, and easy ageing.
RA [96, 97]	\	Activated CD4^+^ T cells (human PB)	Increased	Increased energy demand and enhanced catabolism; resistance to apoptosis. LC3-II was increased and p62 was decreased.
RA [98]	Atg5^f/f^ CD4^Cre^ mice	CD4^+^ T cells (PB)	Decreased	The activation and proliferation of CD4^+^ T cells were reduced. Reduced arthritis incidence and disease severity.
SS [6]	\	SG and T cells (human)	Increased	The level of autophagy was positively correlated with the degree of CD4^+^ T lymphocyte infiltration and the levels of proinflammatory cytokines IL-21 and IL-23.
SS [99, 100]	\	Epithelial cells of the tear membrane and conjunctiva (human)	Increased	The levels of Atg5 and LC3-II/I were increased.
JIA [101]	Treatment with HCQ	CD4^+^ T cells (human PB)	Decreased	CD4^+^ T cell proliferation, cytokine production, and expression of activation markers were reduced.
IBD [56]	Atg16l1^f/f^ CD4^Cre^ mice	T cells	Decreased	The number of CD4^+^, CD8^+^ T cells and Treg cells decreased. Abnormal type II responses to diet and microbiota antigens; Increased Th2 cells.
MS [102]	\	CD4^+^ and CD8^+^ T cells (human)	Increased	Increased Atg5 levels were positively correlated with TNF levels.
MS [103]	PIK3C3^f/f^ CD4^Cre^ mice	T cells (splenocytes)	Decreased	Impaired metabolism and reduced levels of active mitochondria; Failure to initiate autoreactive T cell responses. T cells were unable to differentiate into Th1 cells.
MS [104]	IRGM^-/-^ mice	CD4^+^ T cells (spinal cord)	Decreased	The activation and proliferation of CD4^+^ T cells were decreased, and apoptosis was increased.

SLE, Systemic lupus erythematosus; PB, peripheral blood; CKO, conditional knockout; BAFF, B cell activator; CQ, chloroquine; HCQ, hydroxychloroquine; RA, Rheumatoid arthritis; ATP, adenosine triphosphate; PFKFB3, 6-phosphofructo-2-kinase/fructose-2,6-biphosphatase 3; ROS, reactive oxygen species; SS, Sjögren's syndrome; SG, salivary gland; JIA, Juvenile idiopathic arthritis; IBD, Inflammatory bowel disease; MS, Multiple sclerosis; TNF, tumor necrosis factor; PIK3C3, phosphoinositide-3-Kinase Class 3; IRGM, immune-related GTPase M.

The anti-malarial drugs chloroquine (CQ) and hydroxychloroquine (HCQ), as T-cell non-dependent therapeutic agents, have significant immunomodulatory effects. They act on various molecular and cellular activation pathways of the innate and adaptive immune system and have shown an important role in the clinical management of autoimmune diseases, such as SLE and rheumatoid arthritis (RA). The primary mode of action of CQ and HCQ is to disrupt the endolysosomal system by aggregating in lysosomes. Consequently, the stability of the lysosomal membrane is compromised, resulting in the release of intracellular lysosomal enzymes and the fusion disorder of autophagosome and lysosome, thus blocking autophagy (111, 112). CQ and HCQ, as autophagy inhibitors, have been demonstrated to be effective in the clinical monotherapy of SLE patients (113). It has been reported that SLE mice treated with HCQ exhibited a reduction in the autophagy levels of Th17 and Treg cells, accompanied by a significant reduction in the expression of Th17 cells and IL-17, and a significant increase in the levels of Treg cells and TGF-β. The administration of CQ to SLE mice has been observed to result in a reduction in serum INF-γ, anti-dsDNA IgG, and anti-nuclear IgG levels (93). In SLE patients, the administration of CQ in standard therapeutic doses has been demonstrated to result in a significant reduction in the levels of Th17 cells and serum IL-6, IL-1β, IL-23, and STAT3 (94). In addition to their effects of disrupting the lysosomal system and inhibiting autophagy, CQ and HCQ are capable of producing a number of effects that alter the immune response, such as blocking antigen presentation by DCs, macrophages, B and T cells, and inhibiting the production of pro-inflammatory cytokines such as IL-1, IL-6, IL-17, IFN-α, TNF-α, nitric oxide (NO), and ROS (113). CQ and HCQ can also regulate cellular functions and immune responses by inhibiting protein expression, inducing apoptosis, and inhibiting ion channel activation, which may lead to the treatment of diseases through various immune pathways.

Overall, autophagy levels of T cells were significantly elevated in SLE patients and SLE-susceptible mice, and treatment with autophagy inhibitors improved inflammation levels and clinical symptoms, suggesting that targeting autophagy to modulate overactivated autophagy in T cells appears to be a novel strategy for SLE treatment.

### 4.2 Rheumatoid arthritis

RA, the most common autoimmune arthritis, is primarily characterized by severe inflammation and morphological changes of bone tissue. Persistently active RA leads to joint damage, disability, and a decreased quality of life (96). Cartilage degeneration, synovial hyperplasia, infiltration of synovial fibroblasts (FLS) into cartilage and bone surface, and subchondral bone erosion are all pathological features of RA (114). The pathogenesis of RA is unclear. However, it has been demonstrated that dysregulated immune cells, including Th17 cells, Tfh cells, macrophages, B cells, and FLS are associated with RA (115). Studies have indicated that the APC (such as DC, macrophages, and activated B cells) delivers exogenous antigens or autoantigens to T cells, thereby initiating chronic inflammatory or autoimmune events (116). Furthermore, proinflammatory cytokines and autoantibodies stimulate synovial hyperplasia and bone destruction, which in turn leads to progressive joint destruction (117).

CD4^+^ T cells are considered to be a key driver of the chronic inflammatory response in RA (118). Studies have shown that 6-phosphofructo-2-kinase/fructose-2,6-biphosphatase 3 (PFKFB3) also functions as a positive regulator of autophagy via interaction with Beclin1. However, the expression of glycolytic PFKFB3 is deficient, and intracellular adenosine triphosphate (ATP) production is reduced in naive CD4^+^ T cells from RA patients. Consequently, T cells lack sufficient energy, the generation of reactive oxygen species and the activation of autophagy are insufficient, and T cells are more sensitive to apoptosis and prone to senescence (95). Activated CD4^+^ T cells impose energy requirements, enhance catabolism, and mediate autophagy to maintain homeostasis (96). Consequently, highly activated CD4^+^ T cells isolated from RA patients exhibit increased autophagy activity and relative resistance to apoptotic stimuli (97). The increased LC3-II levels and decreased p62 levels of CD4^+^ T and CD8^+^ T cells in the peripheral blood of RA patients lead to excessive activation of T cells and increased anti-apoptotic ability. However, the anti-apoptotic enhancement of CD4^+^ T cells significantly reverses after autophagy inhibition (97). In a mouse model of collagen-induced arthritis (CIA), autophagy inhibition reduced the incidence and disease severity of arthritis in CIA mice. Furthermore, defective autophagy reduced CD4^+^ T cell activation and proliferation as observed in Atg5^-/-^ CKO mice (98). These studies suggest that autophagy regulates the activation of CD4^+^ T cells, and increased autophagy of activated CD4^+^ T cells can promote the development of RA. At the same time, autophagy can also promote the inflammatory response of RA by regulating inflammatory cytokines and bone destruction.

The results of recent studies have indicated that the administration of HCQ *in vivo* can result in a reduction in the incidence and severity of arthritis in animal models of CIA (98). Furthermore, the clinical application of HCQ has demonstrated efficacy in inhibiting lysosomes, suppressing T cell activity and apoptosis resistance, and reducing the levels of proinflammatory cytokines in RA (98). Consequently, the inhibition of autophagy may represent a promising therapeutic strategy for the treatment of RA.

### 4.3 Sjögren’s syndrome

SS is a chronic systemic autoimmune disease that is characterized by the infiltration of T and B lymphocytes into exocrine glands. The most common symptoms are lesions of salivary glands and lacrimal glands, which present as dry mouth, dry eye, and keratoconjunctivitis (119). The cellular characteristics of SS show that the cell infiltration of the salivary gland (SG) and lacrimal gland (LG) is dominated by CD4^+^ T cells and B cells. In the early stages of the disease, CD4^+^ T cells are primarily infiltrated, suggesting a potential role for these cells in the early immunopathological damage of SS (120). In diseased SG tissues and peripheral blood, CD4^+^ T cells showed an overactivated phenotype (121). This abnormal activation may be caused by T cells interacting with ductal epithelium and infiltrating B cells through costimulatory molecules (CD40/CD40L) and the BAFF axis (6). The current literature indicates that autophagy is a characteristic of T and B cells infiltrating small salivary glands (MSG) in SS patients (122). Evaluation of autophagy markers in SS patients demonstrated that Atg5 and LC3-II/I expression levels in the tear film and conjunctival epithelium are significantly increased (99). The autophagy-related markers (mRNA and protein levels) of T lymphocytes in SG and peripheral blood of SS patients are upregulated, and the level of autophagy is positively correlated with the degree of CD4^+^ T lymphocyte infiltration and the levels of proinflammatory cytokines IL-21 and IL-23. Furthermore, the level of autophagy of T lymphocytes in peripheral blood is positively correlated with the SS disease activity index (6). Another study also demonstrated that both the protein and mRNA levels of Beclin1 and LC3II were increased in peripheral blood CD4^+^ T cells of patients with SS (100). As with the therapeutic effect of HCQ, human umbilical cord mesenchymal stem cell-derived exosomes (UCMSC-Exos) have been shown to reduce the levels of autophagy markers. It restored the balance between Th1/Th2 and Th17/Treg cells and reduced the expression levels of inflammatory factors, including INFγ, TNF-α, IL-6, IL-17A, and IL-17F (100). UCMSC-Exos have been demonstrated to inhibit the excessive proliferation of CD4^+^ T cells by inhibiting autophagy, a process which may lead to the development of a new therapeutic drug for SS.

### 4.4 Juvenile idiopathic arthritis

JIA is a chronic arthritis of unknown etiology with onset before the age of 16. Inflammation in joint synovial fluid (SF) is characterized by an increase in autoreactive, highly activated effector T cells, but the exact pathogenesis remains unclear (123). RNA sequencing reveals that autophagy-related genes in CD4^+^CD45RO^+^ T cells are significantly upregulated in SF of JIA patients compared with HC, suggesting that autophagy is disturbed in T cells of JIA-SF (124). It was found that the level of autophagy protein LC3-II/I was increased in T cells in JIA SF. Inhibition of autophagy by HCQ reduced CD4^+^ T cell proliferation, cytokine production, and activation marker expression in JIA SF (101). Thus, it is speculated that the increased autophagy of T cells in JIA SF is not caused by the inflammatory environment, but is likely the result of high activation of T cells to increase intracellular nutrient supply and meet the metabolic requirements of T cell activation. Therefore, inhibition of autophagy may be a promising therapeutic approach to restore T cell homeostasis in JIA SF.

### 4.5 Inflammatory bowel disease

IBD is a chronic inflammation that primarily affects the gastrointestinal tract, including Crohn’s disease (CD) and ulcerative colitis (UC). In recent years, a large number of studies have identified genetic factors for IBD susceptibility. GWAS and meta-analyses have identified more than 150 different loci that influence IBD susceptibility, revealing new pathways in the pathogenesis of the disease (125). IBD is a disease mediated by IL-17a and IL-1β. GWAS identified Atg16L1 and immune-associated GTPase M (IRGM) deletions in CD, and Atg16L1 gene deletionin bone marrow chimeric mice resulted in excessive IL-1β production by macrophages (126), and altered expression of IRGM resulted in defective autophagy in CD (127), which is related to the pathogenesis of CD and may be one of the etiological factors of IBD. It has been reported that autophagy protects intestinal epithelial cells (IECs) from TNF-induced apoptosis, which maintains the integrity of the intestinal barrier and inhibits the development and progression of IBD (128).

Atg16L is involved in the prolongation of autophagosome membrane (126), and mutation of IBD susceptibility gene T300A in the coding region of Atg16L1 can lead to increased degradation of Atg16L1 protein and reduced autophagy, suggesting that reduced autophagy may contribute to the development of IBD. Changes in the autophagy pathway can alter intestinal homeostasis and lead to chronic intestinal inflammation (129). CD4^+^ T cells constitute the largest population of intestinal lymphocytes and are the central mediators of host protective and tolerance responses in the intestinal tract (130). Deletion of T cell Atg16L1 in CKO mice resulted in a dramatic reduction in the number of CD4^+^ T cells and Treg cells in the intestinal lamina propria (LP), a significant reduction in the frequency of CD4^+^ and CD8^+^ T cells in the peripheral lymphoid organs, and an abnormal type II response to dietary and microbiota antigens, leading to the spontaneous development of progressive chronic intestinal inflammation in the mice (56). Furthermore, increased survival of Atg16L1-deficient Th2 cells was observed *in vitro*, suggesting that autophagy may directly inhibit Th2 cell expansion (56). Although Th17 cells play an important role in IBD disease activity and mucosal injury (131), the specific effect of intestinal inflammation on Th17 cell autophagy remains to be investigated. These findings provide new insights into the interplay of autophagy on different T-cell responses in the gut and have important implications for understanding and treating chronic inflammatory diseases.

### 4.6 Multiple sclerosis

MS is a common inflammatory disease of axonal demyelination and progressive neurodegeneration, caused by oligodendrocyte (OLs) dysfunction and apoptosis in the central nervous system (CNS) (132). It is generally believed that MS is caused by self-reactive T cells targeting CNS myelin (133). T cells penetrate the CNS through the blood-brain barrier, attack the myelin sheath, and initiate chronic inflammatory responses, resulting in the loss of axons and neurons (134).

Some studies have shown that autophagy is directly involved in the disease progression of MS or experimental autoimmune encephalomyelitis (EAE). For example, clonal proliferation of T cells in damaged cerebrospinal fluid (CSF) has been detected in the brains of EAE and active relapse-remitting MS patients (135). And Atg5 and IRGM-1 increased and Atg16L2 decreased in T cells (136). In MS patients, Atg5 mRNA and protein levels are upregulated in CD4^+^ and CD8^+^ T cells at sites of inflammatory infiltration, and Atg5 mRNA levels are positively correlated with TNF levels. This upregulation of autophagy may lead to improved survival rates and active metabolic status of T cells. It may also suggest that Atg5 may be involved in the proinflammatory effect of T cells in MS patients and not affected by autophagy (102). Similar to MS patients, increased expression of the autophagy gene Atg5 is associated with immune-mediated myelin injury in EAE models (137). In contrast, CD4+ and CD8+ T cells from bone marrow chimeric knockout mice with Atg5 failed to proliferate efficiently upon TCR activation (50). These results suggested that prolonged T cell survival and increased T cell proliferation contribute to the recurrence and progression of the disease, suggesting that the overexpression of Atg5 interferes with the abnormal adaptive immune response that leads to the disease (137). However, whether and how the upregulation of Atg5 affects T cell function in MS patients remains to be further studied. Another study found elevated levels of Parkin (a ligase necessary for mitophagy) in serum and CSF in MS patients (102). However, there is still a lack of strong evidence that dysfunctional mitophagy is a disease mechanism of MS.

One successful therapeutic strategy for EAE is silencing over-activated T cells by either inducing T cell apoptosis or blocking T cell activation. Inhibition of CD4^+^ T cell autophagy by T cell-conditioned knockdown of Beclin-1 has been shown to be protective in EAE (138); as a T cell-dependent therapeutic agent, rapamycin can inhibit the initiation of autophagy by inhibiting mTORC1, which in turn inhibits T cell proliferation and activation, reduces the intensity of the immune response, and alleviates relapsing-remitting EAE (134). In a mouse model of EAE with T-cell conditional knockout of the autophagy-associated protein phosphatidylinositol 3-kinase (3PIK3C3/hVPS34), the mice were resistant to EAE, as evidenced by impaired metabolism and reduced levels of active mitochondria, as well as the inability of the mice to mount an autoreactive T-cell response and the inability of the T cells to differentiate into Th1 cells (103). Knockdown of IRGM attenuated the onset and progression of EAE by inhibiting the expansion of activated CD4^+^ T cells and promoting apoptosis (104). DCs specifically conditioned to knock down Atg7 ameliorated the clinical disease severity of actively induced EAE, and DCs conditioned to knock down Atg5 (including CNS DCs) were less capable of stimulating brain-derived CD4^+^ T cells upon uptake of antigenic substances of damaged oligodendrocyte origin, significantly reduced CD4^+^ T cell accumulation in the CNS, and adequately transferred EAE in overtly protected mice from morbidity (139, 140). These results suggest that inhibition of autophagy and T-cell proliferation is beneficial for MS and EAE.

## 5 Conclusion

In conclusion, autophagy plays an important regulatory role in T lymphocyte-mediated autoimmune diseases. Therefore, it is necessary to understand the specific role of autophagy in the development, activation, proliferation, and differentiation of immune cells, and to regulate the function of immune cells by regulating autophagy, to provide an important theoretical basis for the treatment of autophagy-related immune diseases. Of note, autophagy is regulated by multiple protein kinases, especially during autophagy initiation and autolysosome formation. Therefore, many small-molecule compounds regulate autophagy to treat human diseases. The first drugs approved by the FDA to inhibit autophagy, CQ and HCQ, have been applied to clinical practice and produced promising results. Since protein kinases such as AMPK, ULK1, and PI3K/Akt/mTOR play an essential role in autophagy, increasing small molecules that regulate autophagy-related protein kinases are being designed and produced, including PI3K inhibitors, Akt inhibitors, ULK1 activators, and ERK inhibitors, broadening the range of targeted drugs. Therefore, it will be important to selectively target therapeutic agents to specific cell types (e.g., T cells) to target the autophagy pathway.

If targeted modulation of autophagy becomes a viable treatment for autoimmune diseases, there will be additional challenges in developing selective autophagy modulators with little crosstalk with other targets and in addressing drug resistance. Although people in autophagy have made significant progress, it is still unknown to the molecular mechanism of autophagy, involved in the signal process and the participation of autophagy in various disease states. Therefore, future research will aim to elucidate the molecular regulatory mechanism and signaling pathways of autophagy of autophagy in each autoimmune disease and develop highly specific and safe targeted drugs, which will be of great significance for clinical application to identify reliable treatment strategies and monitoring tools.

## Author contributions

XZ: Writing – original draft, Writing – review & editing. DM: Supervision, Writing – review & editing. BY: Resources, Writing – review & editing. YW: Resources, Writing – review & editing. LZ: Supervision, Writing – review & editing.

## References

[1] Zhang XM Liu CY Shao ZH. Advances in the role of helper T cells in autoimmune diseases. Chin Med J (Engl). (2020) 133:968–74. doi: 10.1097/CM9.0000000000000748 PMC7176439 32187054

[2] Ravikumar B Sarkar S Davies JE Futter M Garcia-Arencibia M Green-Thompson ZW. Regulation of mammalian autophagy in physiology and pathophysiology. Physiol Rev. (2010) 90:1383–435. doi: 10.1152/physrev.00030.2009 20959619

[3] Yang Z Goronzy JJ Weyand CM. Autophagy in autoimmune disease. J Mol Med (Berl). (2015) 93:707–17. doi: 10.1007/s00109-015-1297-8 PMC4486076 26054920

[4] Li C Capan E Zhao Y Zhao J Stolz D Watkins SC. Autophagy is induced in CD4+T cells and important for the growth factor-withdrawal cell death. J Immunol. (2006) 177:5163–8. doi: 10.4049/jimmunol.177.8.5163 17015701

[5] Gerland LM Genestier L Peyrol S Michallet MC Hayette S Urbanowicz I. Autolysosomes accumulate during in *vitro* CD8+ T-lymphocyte aging and may participate in induced death sensitization of senescent cells. Exp Gerontol. (2004) 39:789–800. doi: 10.1016/j.exger.2004.01.013 15130673

[6] Alessandri C Ciccia F Priori R Astorri E Guggino G Alessandro R. CD4 T lymphocyte autophagy is upregulated in the salivary glands of primary Sjögren’s syndrome patients and correlates with focus score and disease activity. Arthritis Res Ther. (2017) 19:178. doi: 10.1186/s13075-017-1385-y 28743286 PMC5526255

[7] Dikic I Elazar Z. Mechanism and medical implications of mammalian autophagy. Nat Rev Mol Cell Biol. (2018) 19:349–64. doi: 10.1038/s41580-018-0003-4 29618831

[8] Vargas JNS Hamasaki M Kawabata T. The mechanisms and roles of selective autophagy in mammals. Nat Rev Mol Cell Biol. (2023) 24:167–85. doi: 10.1038/s41580-022-00542-2 36302887

[9] Gros F Muller S. The role of lysosomes in metabolic and autoimmune diseases. Nat Rev Nephrol. (2023) 19:366–83. doi: 10.1038/s41581-023-00692-2 36894628

[10] Cuervo AM Wong E. Chaperone-mediated autophagy: roles in disease and aging. Cell Res. (2014) 24:92–104. doi: 10.1038/cr.2013.153 24281265 PMC3879702

[11] Li WW Li J Bao JK. Microautophagy: lesser-known self-eating. Cell Mol Life Sci. (2012) 69:1125–36. doi: 10.1007/s00018-011-0865-5 PMC11114512 22080117

[12] Mizushima N Levine B Cuervo AM. Autophagy fights disease through cellular self-digestion. Nature. (2008) 451:1069–75. doi: 10.1038/nature06639 PMC2670399 18305538

[13] Reggiori F. Membrane origin for autophagy. Curr Top Dev Biol. (2006) 74:1–30. doi: 10.1016/S0070-2153(06)74001-7 16860663 PMC7112310

[14] Yang S Long LH Li D Zhang JK Jin S Wang F. β-Guanidinopropionic acid extends the lifespan of Drosophila melanogaster via an AMP-activated protein kinase-dependent increase in autophagy. Aging Cell. (2015) 14:1024. doi: 10.1111/acel.12371 26120775 PMC4693457

[15] Estrada-Navarrete G Cruz-Mireles N Lascano R Alvarado-Affantranger X Hernández-Barrera A Barraza A. An autophagy-related kinase is essential for the symbiotic relationship between Phaseolus vulgaris and both rhizobia and arbuscular mycorrhizal fungi. Plant Cell. (2016) 28:2326–41. doi: 10.1105/tpc.15.01012 PMC5059792 27577790

[16] Mizushima N Yoshimori T Ohsumi Y. The role of Atg proteins in autophagosome formation. Annu Rev Cell Dev Biol. (2011) 27:107–32. doi: 10.1146/annurev-cellbio-092910-154005 21801009

[17] Lippai M Löw P. The role of the selective adaptor p62 and ubiquitin-like proteins in autophagy. BioMed Res Int. (2014) 2014:832704. doi: 10.1155/2014/832704 25013806 PMC4075091

[18] Galluzzi L Baehrecke EH Ballabio A Boya P Bravo-San Pedro JM Cecconi F. Molecular definitions of autophagy and related processes. EMBO J. (2017) 36:1811–36. doi: 10.15252/embj.201796697 PMC5494474 28596378

[19] Lee Y Weihl CC. Regulation of SQSTM1/p62 via UBA domain ubiquitination and its role in disease. Autophagy. (2017) 13:1615–6. doi: 10.1080/15548627.2017.1339845 PMC5612413 28812433

[20] Settembre C Di Malta C Polito VA Arencibia MG Vetrini F Erdin S. TFEB links autophagy to lysosomal biogenesis. Science. (2011) 332:1429–33. doi: 10.1126/science.1204592 PMC3638014 21617040

[21] Klionsky DJ Abdel-Aziz AK Abdelfatah S Abdellatif M Abdoli A Abel S. Guidelines for the use and interpretation of assays for monitoring autophagy. Autophagy. (2021) 17:1–382. doi: 10.1080/15548627.2020.1797280 33634751 PMC7996087

[22] Kim J Guan KL. mTOR as a central hub of nutrient signalling and cell growth. Nat Cell Biol. (2019) 21:63–71. doi: 10.1038/s41556-018-0205-1 30602761

[23] Salminen A Kaarniranta K Kauppinen A. Beclin1 interactome controls the crosstalk between apoptosis, autophagy and inflammasome activation: impact on the aging process. Ageing Res Rev. (2013) 12:520–34. doi: 10.1016/j.arr.2012.11.004 23220384

[24] Lee XC Werner E Falasca M. Molecular mechanism of autophagy and its regulation by cannabinoids in cancer. Cancers(Basel). (2021) 13:1211. doi: 10.3390/cancers13061211 33802014 PMC7999886

[25] Egan DF Shackelford DB Mihaylova MM Gelino S Kohnz RA Mair W. Phosphorylation of ULK1 (hATG1) by AMP-activated protein kinase connects energy sensing to mitophagy. Science. (2011) 331:456–61. doi: 10.1126/science.1196371 PMC3030664 21205641

[26] Jang M Park R Kim H Namkoong S Jo D Huh YH. AMPK contributes to autophagosome maturation and lysosomal fusion. Sci Rep. (2018) 8:12637. doi: 10.1038/s41598-018-30977-7 30140075 PMC6107659

[27] Reedquist KA Ludikhuize J Tak PP. Phosphoinositide 3-kinase signalling and FoxO transcription factors in rheumatoid arthritis. Biochem Soc Trans. (2006) 34:727–30. doi: 10.1042/BST0340727 17052183

[28] Jung CH Ro SH Cao J. mTOR regulation of autophagy. FFBS Lett. (2010) 584:1287–95. doi: 10.1016/j.febslet.2010.01.017 PMC2846630 20083114

[29] Kim J Kundu M Viollet B. AMPK and mTOR regulate autophagy through direct phosphorylation of Ulk1. Nat Cell Biol. (2011) 13:132–41. doi: 10.1038/ncb2152 PMC3987946 21258367

[30] Jewell JL Russell RC Guan KL. Amino acid signalling upstream of mTOR. Nat Rev Mol Cell Biol. (2013) 14:133–9. doi: 10.1038/nrm3522 PMC3988467 23361334

[31] Xiang H Zhang J Lin C Zhang L Liu B Ouyang L. Targeting autophagy-related protein kinases for potential therapeutic purpose. Acta Pharm Sin B. (2020) 10:569–81. doi: 10.1016/j.apsb.2019.10.003 PMC7161711 32322463

[32] Raudenska M Balvan J Masarik M. Crosstalk between autophagy inhibitors and endosome-related secretory pathways: a challenge for autophagy-based treatment of solid cancers. Mol Cancer. (2021) 20:140. doi: 10.1186/s12943-021-01423-6 34706732 PMC8549397

[33] Nicklin P Bergman P Zhang B Triantafellow E Wang H Nyfeler B. Bidirectional transport of amino acids regulates mtor and autophagy. Cell. (2009) 136:521–34. doi: 10.1016/j.cell.2008.11.044 PMC3733119 19203585

[34] Garcia-Martinez JM Alessi DR. mTOR complex 2 (mTORC2) controls hydrophobic motif phosphorylation and activation of serum- and glucocorticoid-induced protein kinase 1 (SGK1). Biochem. (2008) 416:375–85. doi: 10.1042/BJ20081668 18925875

[35] Wang R Wang G. Protein modification and autophagy activation. Adv Exp Med Biol. (2019) 1206:237–59. doi: 10.1007/978-981-15-0602-4_12 31776989

[36] Brunet A Bonni A Zigmond MJ Lin MZ Juo P Hu LS. Akt promotes cell survival by phosphorylating and inhibiting a Forkhead transcription factor. Cell. (1999) 96:857–68. doi: 10.1016/S0092-8674(00)80595-4 10102273

[37] Ying W. Hongbing Z.Regulation of autophagy by mTOR signaling pathway. Adv Exp Med Biol. (2019) 1206:67–83. doi: 10.1007/978-981-15-0602-4_3 31776980

[38] Cao Y Klionsky DJ. Physiological functions of Atg6/Beclin1: a unique autophagy-related protein. Cell Res. (2007) 17:839–49. doi: 10.1038/cr.2007.78 17893711

[39] Sahni S Merlot AM Krishan S. Gene of the month: BECN1. J Clin Pathol. (2014) 67:656–60. doi: 10.1136/jclinpath-2014-202356 24811486

[40] Zalckvar E Berissi H Mizrachy L Idelchuk Y Koren I Eisenstein M. DAP-kinase-mediated phosphorylation on theBH3 domain of beclin1 promotes dissociation of beclin1 from Bcl-XL and induction of autophagy. EMBO Rep. (2009) 10:285–92. doi: 10.1038/embor.2008.246 PMC2658558 19180116

[41] Furuya T Kim M Lipinski M Li J Kim D Lu T. Negative regulation of Vps34 by Cdk mediated phosphorylation. Mol Cell. (2010) 38:500–11. doi: 10.1016/j.molcel.2010.05.009 PMC2888511 20513426

[42] Keller CW Adamopoulos IE Lünemann JD. Autophagy pathways in autoimmune diseases. J Autoimmun. (2023) 136:103030. doi: 10.1016/j.jaut.2023.103030 37001435 PMC10709713

[43] Boya P Codogno P Rodriguez-Muela N. Autophagy in stem cells: Repair, remodelling and metabolic reprogramming. Development. (2018) 145:dev146506. doi: 10.1242/dev.146506 29483129

[44] Nedjic J Aichinger M Emmerich J. Autophagy in thymic epithelium shapes the T-cell repertoire and is essential for tolerance. Nature. (2008) 455:396–400. doi: 10.1038/nature07208 18701890

[45] Schmid D Pypaert M Münz C. Antigen-loading compartments for major histocompatibility complex class II molecules continuously receive input from autophagosomes. Immunity. (2007) 26:79–92. doi: 10.1016/j.immuni.2006.10.018 17182262 PMC1805710

[46] Hei JK Joanna BS Satoshi I. Ubiquitination of MHC class II by march-I regulates dendritic cell fitness. J Immunol. (2021) 206:494–504. doi: 10.4049/jimmunol.2000975 33318291 PMC9169697

[47] Mcleod IX Jia W He Y. The contribution of autophagy to lymphocyte survival and homeostasis. Immunol Rev. (2012) 249:195–204. doi: 10.1111/j.1600-065X.2012.01143.x 22889223 PMC3602971

[48] Jia W Pua HH Li QJ. Autophagy regulates endoplasmic reticulum homeostasis and calcium mobilization in T lymphocytes. J Immunol. (2011) 186:1564–74. doi: 10.4049/jimmunol.1001822 PMC3285458 21191072

[49] Hubbard VM Valdor R Patel B Singh R Cuervo AM Macian F. Macroautophagy regulates energy metabolism during effector T cell activation. J Immunol. (2010) 185:7349–57. doi: 10.4049/jimmunol.1000576 PMC3046774 21059894

[50] Pua HH Dzhagalov I Chuck M. A critical role for the autophagy gene Atg5 in T cell survival and proliferation. J Exp Med. (2007) 204:25–31. doi: 10.1084/jem.20061303 17190837 PMC2118420

[51] Jia W He YW. Temporal regulation of intracellular organelle homeostasis in T lymphocytes by autophagy. J Immunol. (2011) 186:5313–22. doi: 10.4049/jimmunol.1002404 21421856

[52] He W Xiong W Xia X. Autophagy regulation of mammalian immune cells. Adv Exp Med Biol. (2019) 1209:7–22. doi: 10.1007/978-981-15-0606-2_2 31728862

[53] Botbol Y Patel B Macian F. Common γ-chaincytokine signaling is required for macroautophagy induction during CD4+ T cell activation. Autophagy. (2015) 11:1864–77. doi: 10.1080/15548627.2015.1089374 PMC4824584 26391567

[54] Macian F. Autophagy in T cell function and aging. Front Cell Dev Biol. (2019) 7:213. doi: 10.3389/fcell.2019.00213 31632966 PMC6783498

[55] Zhao HY Dong F Li YH Ren X Xia Z Wang Y. Inhibiting ATG5 mediated autophagy to regulate endoplasmic reticulum stress and CD4+ T lymphocyte differentiation: Mechanisms of acupuncture's effects on asthma. BioMed Pharmacother. (2021) 142:112045. doi: 10.1016/j.biopha.2021.112045 34426257

[56] Kabat AM Harrison OJ Riffelmacher T Moghaddam AE Pearson CF Laing A. The autophagy gene Atg16l1 differentially regulates Treg and TH2 cells to control intestinal inflammation. Elife. (2016) 5:e12444. doi: 10.7554/eLife.12444 26910010 PMC4798959

[57] Zhang SF Huang XF Xiu HQ Zhang Z Zhang K Cai J. The attenuation of Th1 and Th17 responses via autophagy protects against methicillin-resistant Staphylococcus aureus-induced sepsis. Microbes Infect. (2021) 23:104833. doi: 10.1016/j.micinf.2021.104833 33930602

[58] Delgoffe GM Kole TP Zheng Y Zarek PE Matthews KL Xiao B. The mTOR kinase differentially regulates effector and regulatory T cell lineage commitment. Immunity. (2009) 30:832–44. doi: 10.1016/j.immuni.2009.04.014 PMC2768135 19538929

[59] Delgoffe GM Pollizzi KN Waickman AT Heikamp E Meyers DJ Horton MR. The kinase mTOR regulates the differentiation of helper T cells through the selective activation of signaling by mTORC1 and mTORC2. Nat Immunol. (2011) 12:295–303. doi: 10.1038/ni.2005 21358638 PMC3077821

[60] Lee K Gudapati P Dragovic S Spencer C Joyce S Killeen N. Mammalian target of rapamycin protein complex 2 regulates differentiation of Th1 and Th2 cell subsets via distinct signaling pathways. Immunity. (2010) 32:743–53. doi: 10.1016/j.immuni.2010.06.002 PMC2911434 20620941

[61] Yang K Shrestha S Zeng H Karmaus PWF Neale G Vogel P. T cell exit from quiescence and differentiation into Th2 cells depend on Raptor-mTORC1-mediated metabolic reprogramming. Immunity. (2013) 39:1043–56. doi: 10.1016/j.immuni.2013.09.015 PMC3986063 24315998

[62] Zeng H Yang K Cloer C Neale G Vogel P Chi H. mTORC1 couples immune signals and metabolic programming to establish T(reg)-cell function. Nature. (2013) 499:485–90. doi: 10.1038/nature12297 PMC3759242 23812589

[63] Michalek RD Gerriets VA Jacobs SR Macintyre AN MacIver NJ Mason EF. Cutting edge: distinct glycolytic and lipid oxidative metabolic programs are essential for effector and regulatory CD4+ T cell subsets. J Immunol. (2011) 186:3299–303. doi: 10.4049/jimmunol.1003613 PMC3198034 21317389

[64] Thaiz RV Cai ZJ Shen YY Dosset M Benoit-Lizon I Martin T. Selective degradation of PU.1 during autophagy represses the differentiation and antitumour activity of TH9 cells. Nat Commun. (2017) 8:559. doi: 10.1038/s41467-017-00468-w 28916785 PMC5602674

[65] He YQ Qiao YL Xu S Jiao WE Yang R Kong YG. Allergen induces CD11c+ dendritic cell autophagy to aggravate allergicrhinitis through promoting immune imbalance. Int Immunopharmacol. (2022) 106:108611. doi: 10.1016/j.intimp.2022.108611 35158226

[66] Swadling L Pallett LJ Diniz MO Baker JM Amin OE Stegmann KA. Human liver memory CD8+ T cells use autophagy for tissue residence. Cell Rep. (2019) 30:687–98. doi: 10.1016/j.celrep.2019.12.050 PMC6988113 31968246

[67] Dowling SD Macian F. Autophagy and T cell metabolism. Cancer Lett. (2018) 419:20–6. doi: 10.1016/j.canlet.2018.01.033 PMC5937942 29339212

[68] Mocholi E Dowling SD Botbol Y Gruber RC Ray AK Vastert S. Autophagy is a tolerance-avoidance mechanism that modulates TCR-mediated signaling and cell metabolism to prevent induction of T cell anergy. Cell Rep. (2018) 24:1136–50. doi: 10.1016/j.celrep.2018.06.065 PMC6109966 30067971

[69] Whang MI Tavares RM Benjamin DI Kattah MG Advincula R Nomura DK. The ubiquitin binding protein TAX1BP1 mediates autophagosome induction and the metabolic transition of activated T cells. Immunity. (2017) 46:405–20. doi: 10.1016/j.immuni.2017.02.018 PMC5400745 28314591

[70] Pua HH Guo J Komatsu M. Autophagy is essential for mitochondrial clearance in mature T lymphocytes. J Immunol. (2009) 182:4046–55. doi: 10.4049/jimmunol.0801143 19299702

[71] Becher J Simula L Volpe E Procaccini C Rocca CL D'Acunzo P. AMBRA1 controls regulatory T-cell differentiation and homeostasis upstream of the FOXO3-FOXP3 axis. Dev Cell. (2018) 47:592–607. doi: 10.1016/j.devcel.2018.11.010 30513302

[72] Qiu G Liu J Cheng Q Wang Q Jing Z Pei Y. Impaired autophagy and defective T cell homeostasis in mice with T cell-specific deletion of receptor for activated C kinase 1. Front Immunol. (2017) 8:575. doi: 10.3389/fimmu.2017.00575 28572806 PMC5435764

[73] Hu L-F. Epigenetic regulation of autophagy. Adv Exp Med Biol. (2019) 1206:221–36. doi: 10.1007/978-981-15-0602-4_11 31776988

[74] Artal-Martinez de Narvajas A Gomez TS Zhang JS Mann AO Taoda Y Gorman JA. Epigenetic regulation of autophagy by the methyltransferase G9a. Mol Cell Biol. (2013) 33:3983–93. doi: 10.1128/MCB.00813-13 PMC3811684 23918802

[75] Ceccarelli F Perricone C Colasanti T Massaro L Cipriano E Pendolino M. Anti-carbamylated protein antibodies as a new biomarker of erosive joint damage in systemic lupus erythematosus. Arthritis Res Ther. (2018) 20:126. doi: 10.1186/s13075-018-1622-z 29898764 PMC6001021

[76] Yang YF Wang CM Hsiao IH Liu YL Lin WH Lin CL. Peptidylarginine deiminase 2 promotes T helper 17-like T cell activation and activated T cell-autonomous death (ACAD) through an endoplasmic reticulum stress and autophagy coupling mechanism. Cell Mol Biol Lett. (2022) 27:19. doi: 10.1186/s11658-022-00312-0 35236296 PMC8903576

[77] Ireland JM Unanue ER. Autophagy in antigen-presenting cells results in presentation of citrullinated peptides to CD4 T cells. J Exp Med. (2011) 208:2625–32. doi: 10.1084/jem.20110640 PMC3244027 22162830

[78] Pierdominici M Vomero M Barbati C Colasanti T Maselli A Vacirca D. Role of autophagy in immunity and autoimmunity, with a special focus on systemic lupus erythematosus. FASEB J. (2012) 26:1400–12. doi: 10.1096/fj.11-194175 22247332

[79] Lo MS Tsokos GC. Recent developments in systemic lupus erythematosus pathogenesis and applications for therapy. Curr Opin Rheumatol. (2018) 30:222–8. doi: 10.1097/BOR.0000000000000474 PMC6050980 29206660

[80] Zhou XJ Lu XL Lv JC Yang HZ Qin LX Zhao MH. Genetic association of PRDM1-ATG5 intergenic region and autophagy with systemic lupus erythematosus in a Chinese population. Ann Rheum Dis. (2011) 70:1330–7. doi: 10.1136/ard.2010.140111 21622776

[81] Zhang YM Zhou XJ Cheng FJ Qi YY Hou P Zhao MH. Autophagy-related gene LRRK2 is likely a susceptibility gene for systemic lupus erythematosus in northern Han Chinese. Oncotarget. (2017) 8:13754–61. doi: 10.18632/oncotarget.v8i8 PMC5355135 28099919

[82] Yang W Tang H Zhang Y Tang X Zhang J Sun L. Meta-analysis followed by replication identifies loci in or near CDKN1B, TET3, CD80, DRAM1, and ARID5B as associated with systemic lupus erythematosus in Asians. Am J Hum Genet. (2013) 92:41–51. doi: 10.1016/j.ajhg.2012.11.018 23273568 PMC3542470

[83] Zhou XJ Nath SK Qi YY Cheng FJ Yang HZ Zhang Y. Brief Report: identification of MTMR3 as a novel susceptibility gene for lupus nephritis in northern Han Chinese by shared-gene analysis with IgA nephropathy. Arthritis Rheumatol. (2014) 66:2842–8. doi: 10.1002/art.38749 PMC4180767 24943867

[84] Freedman BI Langefeld CD Andringa KK Croker JA Williams AH Garner NE. End-stage renal disease in African Americans with lupus nephritis is associated with APOL1. Arthritis Rheumatol. (2014) 66:390–6. doi: 10.1002/art.38220 PMC4002759 24504811

[85] Lopez P Alonso-Perez E Rodriguez-Carrio J. Influence of Atg5 mutation in SLE depends on functional IL-10 genotype. PloS One. (2013) 8:e78756. doi: 10.1371/journal.pone.0078756 24205307 PMC3799636

[86] Moulton VR Tsokos GC. Abnormalities of T cell signaling in systemic lupus erythematosus. Arthritis Res Ther. (2011) 13:207. doi: 10.1186/ar3251 21457530 PMC3132009

[87] Deretic V Saitoh T Akira S. Autophagy in infection, inflammation and immunity. Nat Rev Immunol. (2013) 13:722–37. doi: 10.1038/nri3532 PMC5340150 24064518

[88] La Cava A. Lupus and T cells. Lupus. (2009) 18:196–201. doi: 10.1177/0961203308098191 19213856

[89] Caielli S Cardenas J de Jesus AA Baisch J Walters L Blanck JP. Erythroid mitochondrial retention triggers myeloid-dependent type I interferon in human SLE. Cell. (2021) 184:4464–79. doi: 10.1016/j.cell.2021.07.021 PMC8380737 34384544

[90] Wu ZZ Zhang JJ Gao CC Zhao M Liu SY Gao GM. Expression of autophagy related genes mTOR, Becline-1, LC3 and p62 in the peripheral blood mononuclear cells of systemic lupus erythematosus. Am J Clin Exp Immunol. (2017) 6:1–8. 28123902 PMC5259582

[91] Alessandri C Barbati C Vacirca D Piscopo P Confaloni A Sanchez M. T lymphocytes from patients with systemic lupus erythematosus are resistant to induction of autophagy. FASEB J. (2012) 26:4722–32. doi: 10.1096/fj.12-206060 PMC3475261 22835828

[92] Liu CJ Tang SJ Chou CC. *In Vivo* Suppression of Autophagy via Lentiviral shRNA Targeting Atg5 Improves Lupus-Like Syndrome. BioMed Res Int. (2020) 2020:8959726. doi: 10.1155/2020/8959726 32462028 PMC7212279

[93] An N Chen Y Wang C Yang C Wu ZH Xue J. Chloroquine autophagic inhibition rebalances Th17/Treg-mediated immunity and ameliorates systemic lupus erythematosus. Cell Physiol Biochem. (2017) 44:412–22. doi: 10.1159/000484955 29141242

[94] Wen Z Xu L Xu W. Detection of dynamic frequencies of Th17 cells and their associations with clinical parameters in patients with systemic lupus erythematosus receiving standard therapy. Clin Rheumatol. (2014) 33:1451–8. doi: 10.1007/s10067-014-2656-5 24810699

[95] Yang Z Fujii H Shalini V. Phosphofructokinase deficiency impairs ATP generation, autophagy, and redox balance in rheumatoid arthritis T cells. J Exp Med. (2013) 210:2119–34. doi: 10.1084/jem.20130252 PMC3782046 24043759

[96] Dai YJ Hu SX. Recent insights into the role of autophagy in the pathogenesis of rheumatoid arthritis. Rheumatology. (2016) 55:403–10. doi: 10.1093/rheumatology/kev337 26342228

[97] Chen YM Chang CY Chen HH Hsieh CW Tang KT Yang MC. Association between autophagy and inflammation in patients with rheumatoid arthritis receiving biologic therapy. Arthritis Res Ther. (2018) 20:268. doi: 10.1186/s13075-018-1763-0 30518408 PMC6280483

[98] van Loosdregt J Rossetti M Spreafico R Moshref M Olmer M Williams GW. Increased autophagy in CD4+T cells of rheumatoid arthritis patients results in T-cell hyperactivation and apoptosis resistance. Eur J Immunol. (2016) 46:2862–70. doi: 10.1002/eji.201646375 PMC5138112 27624289

[99] Byun YS Lee HJ Shin S. Elevation of autophagy markers in Sjogren syndrome dry eye. Sci Rep. (2017) 7:17280. doi: 10.1038/s41598-017-17128-0 29222450 PMC5722946

[100] Ma D Wu Z Zhao X Zhu X An Q Wang Y. Immunomodulatory effects of umbilical mesenchymal stem cell-derived exosomes on CD4+ T cells in patients with primary Sjögren's syndrome. Inflammopharmacology. (2023) 31:1823–38. doi: 10.1007/s10787-023-01189-x PMC10352432 37012581

[101] Peeters JGC de Graeff N Lotz M Albani S Roock S Loosdregt J. Increased autophagy contributes to the inflammatory phenotype of juvenile idiopathic arthritis synovial fluid T cells. Rheumatology. (2017) 56:1694–9. doi: 10.1093/rheumatology/kex227 PMC5850277 28957547

[102] Patergnani S Castellazzi M Bonora M Marchi S Casetta I Pugliatti M. Autophagy and mitophagy elements are increased in body fluids of multiple sclerosis-affected individuals. J Neurol Neurosurg Psychiatry. (2018) 89:439–41. doi: 10.1136/jnnp-2017-316234 28866627

[103] Yang G Song W Postoak JL Chen J Martinez J Zhang J. Autophagy-related protein PIK3C3/VPS34 controls T cell metabolism and function: PIK3C3/VPS34 in T cell metabolism and function. Autophagy. (2021) 17:1193–204. doi: 10.1080/15548627.2020.1752979 PMC8143264 32268825

[104] Xu H Wu Z Fang F Guo L Chen D Chen JX. Genetic deficiency of Irgm1 (LRG-47) suppresses induction of experimental autoimmune encephalomyelitis by promoting apoptosis of activated CD4+T cells. FASEB J. (2010) 24:1583–92. doi: 10.1096/fj.09-137323 PMC2879948 20056715

[105] Ciuffreda L Di Sanza C Incani UC. The mTOR pathway: a new target in cancer therapy. Curr Cancer Drug Targets. (2010) 10:484–95. doi: 10.2174/156800910791517172 20384580

[106] Levine B Sinha S Kroemer G. Bcl-2 family members: dual regulators of apoptosis and autophagy. Autophagy. (2008) 4:600–6. doi: 10.4161/auto.6260 PMC2749577 18497563

[107] Djavaheri-Mergny M Amelotti M Mathieu J Besançon F Bauvy C Souquèr S. NF-κB activation represses tumor necrosis factor-α-induced autophagy. J Biol Chem. (2006) 281:30373–82. doi: 10.1074/jbc.M602097200 16857678

[108] Winslow AR Chen CW Corrochano S Acevedo-Arozena A Gordon DE Peden AA. α-Synuclein impairs macroautophagy: implications for Parkinson’s disease. J Cell Biol. (2010) 190:1023–37. doi: 10.1083/jcb.201003122 PMC3101586 20855506

[109] Walsh CM Edinger AL. The complex interplay between autophagy, apoptosis, and necrotic signals promotes T-cell homeostasis. Immunol Rev. (2010) 236:95–109. doi: 10.1111/j.1600-065X.2010.00919.x 20636811 PMC2966323

[110] Powell JD Delgoffe GM. The mammalian target of rapamycin: linking T cell differentiation, function, and metabolism. Immunity. (2010) 33:301–11. doi: 10.1016/j.immuni.2010.09.002 PMC2962404 20870173

[111] Mauthe M Orhon I Rocchi C Zhou X Luhr M Hijlkema KJ. Chloroquine inhibits autophagic flux by decreasing autophagosome-lysosome fusion. Autophagy. (2018) 14:1435–55. doi: 10.1080/15548627.2018.1474314 PMC6103682 29940786

[112] Circu M Cardelli J Barr MP O'Byrne K Mills G El-Osta H. Modulating lysosomal function through lysosome membrane permeabilization or autophagy suppression restores sensitivity to cisplatin in refractory non-small-cell lung cancer cells. PloS One. (2017) 12:e0184922. doi: 10.1371/journal.pone.0184922 28945807 PMC5612465

[113] Martinez GP Zabaleta ME Di Giulio C. The role of chloroquine and hydroxychloroquine in immune regulation and diseases. Curr Pharm Des. (2020) 26:4467–84. doi: 10.2174/1381612826666200707132920 32634079

[114] Pope RM. Apoptosis as a therapeutic tool in rheumatoid arthritis. Nat Rev Immunol. (2002) 2:527–35. doi: 10.1038/nri846 12094227

[115] Firestein GS. Evolving concepts of rheumatoid arthritis. Nature. (2003) 423:356–61. doi: 10.1038/nature01661 12748655

[116] Choy E. Understanding the dynamics: pathways involved in the pathogenesis of rheumatoid arthritis. Rheumatology. (2012) 51:3–11. doi: 10.1093/rheumatology/kes113 22718924

[117] Schett G Firestein GS. Mr Outside and Mr Inside: classic and alternative views on the pathogenesis of rheumatoid arthritis. Ann Rheum Dis. (2010) 69:787–9. doi: 10.1136/ard.2009.121657 20299352

[118] Weyand CM Fujii H Shao L. Rejuvenating the immune system in rheumatoid arthritis. Nat Rev Rheumatol. (2009) 5:583–8. doi: 10.1038/nrrheum.2009.180 19798035

[119] Srivastava A Makarenkova HP. Innate immunity and biological therapies for the treatment of Sjögren’s syndrome. Int J Mol Sci. (2020) 21:9172. doi: 10.3390/ijms21239172 33271951 PMC7730146

[120] Verstappen GM Kroese FGM Bootsma H. T cells in primary Sjogren’s syndrome: targets for early intervention. Rheumatol. (2019) 60:3088–98. doi: 10.1093/rheumatology/kez004 PMC8516500 30770920

[121] Singh N Cohen PL. The T cell in Sjogren's syndrome: force majeure, not spectateur. J Autoimmun. (2012) 39:229–33. doi: 10.1016/j.jaut.2012.05.019 PMC3428488 22709856

[122] ColaFrancesco S Vomero M Iannizzotto V Minniti A Barbati C Arienzo F. Autophagy-occurs in lymphocytes infiltrating Sjögren’s syndrome minor salivary glands and correlates with histological severity of salivary gland lesions. Arthritis Res Ther. (2020) 22:238. doi: 10.1186/s13075-020-02317-6 33050949 PMC7557086

[123] Prakken B Albani S Martini A. Juvenile idiopathic arthritis. Lancet. (2011) 377:2138–49. doi: 10.1016/S0140-6736(11)60244-4 21684384

[124] Peeters JGC Vervoort SJ Tan SC Mijnheer G Roock S Vastert SJ. Inhibition of super-enhancer activity in autoinflammatory site-derived T cells reduces disease-associated gene expression. Cell Rep. (2015) 12:1986–96. doi: 10.1016/j.celrep.2015.08.046 26387944

[125] Van Limbergen J Radford-Smith G Satsangi J. Advances in IBD genetics. Nat Revi Gastroenterol Hepatol. (2014) 11:372–85. doi: 10.1038/nrgastro.2014.27 24614343

[126] Saitoh T Fujita N Jang MH Uematsu S Yang BG Satoh T. Loss of the autophagy protein Atg16L1 enhances endotoxin-induced IL-1beta production. Nature. (2008) 456:264–8. doi: 10.1038/nature07383 18849965

[127] McCarroll SA Huett A Kuballa P Chilewski SD Landry A Goyette P. Deletion polymorphism upstream of IRGM associated with altered IRGM expression and Crohn’s disease. Nat Genet. (2008) 40:1107–12. doi: 10.1038/ng.215 PMC2731799 19165925

[128] Pott J Kabat AM Maloy KJ. Intestinal Epithelial Cell Autophagy Is Required to Protect against TNF-Induced Apoptosis during Chronic Colitis in Mice. Cell Host Microbe. (2018) 23:191–202. doi: 10.1016/j.chom.2017.12.017 29358084

[129] Murthy A Li Y Peng I Reichelt M Katakam AK Noubade R. A Crohn’s disease variant in Atg16l1 enhances its degradation by caspase 3. Nature. (2014) 506:456–62. doi: 10.1038/nature13044 24553140

[130] Shale M Schiering C Powrie F. CD4(+) T-cell subsets in intestinal inflammation. Immunol Rev. (2013) 252:164–82. doi: 10.1111/imr.12039 PMC3736165 23405904

[131] Globig AM Hennecke N Martin B Seidl M Ruf G Hasselblatt P. Comprehensive intestinal T helper cell profiling reveals specific accumulation of IFN-γ+IL-17+coproducing CD4+ T cells in active inflammatory bowel disease. Inflammation Bowel Dis. (2014) 20:2321–9. doi: 10.1097/MIB.0000000000000210 25248005

[132] Attfield KE Jensen LT Kaufmann M. The immunology of multiple sclerosis. Nat Rev Immunol. (2022) 22:734–50. doi: 10.1038/s41577-022-00718-z 35508809

[133] Glass CK Saijo K Winner B. Mechanisms underlying inflammation in neurodegeneration. Cell. (2010) 140:918–34. doi: 10.1016/j.cell.2010.02.016 PMC2873093 20303880

[134] Liang PZ Le WD. Role of autophagy in the pathogenesis of multiple sclerosis. Neurosci Bull. (2015) 31:435–44. doi: 10.1007/s12264-015-1545-5 PMC5563716 26254059

[135] Jacobsen M Cepok S Quak E Happel M Gaber R Ziegler A. Oligoclonal expansion of memory CD8+ T cells in cerebrospinal fluid from multiple sclerosis patients. Brain. (2002) 125:538–50. doi: 10.1093/brain/awf059 11872611

[136] Igci M Baysan M Yigiter R Ulasli M Geyik S Bayraktar R. Gene expression profiles of autophagy-related genes in multiple sclerosis. Gene. (2016) 588:38–46. doi: 10.1016/j.gene.2016.04.042 27125224

[137] Alirezaei M Fox HS Flynn CT Moore CS Hebb ALO Frausto RF. Elevated Atg5 expression in autoimmune demyelination and multiple sclerosis. Autophagy. (2009) 5:152–8. doi: 10.4161/auto.5.2.7348 PMC2779564 19066443

[138] Kovacs JR Li C Yang Q Li G Garcia IG Ju S. Autophagy promotes T-cell survival through degradation of proteins of the cell death machinery. Cell Death Differ. (2012) 19:144–52. doi: 10.1038/cdd.2011.78 PMC3252822 21660048

[139] Keller CW Sina C Kotur MB Ramelli G Mundt S Quast I. ATG-dependent phagocytosis in dendritic cells drives myelin-specific CD4+ T cell pathogenicity during CNS inflammation. Proc Natl Acad Sci U.S.A. (2017) 114:E11228–37. doi: 10.1073/pnas.1713664114 PMC5748192 29233943

[140] Keller CW Lünemann JD. Noncanonical autophagy in dendritic cells triggers CNS autoimmunity. Autophagy. (2018) 14:560–1. doi: 10.1080/15548627.2018.1427397 PMC5915019 29368985

